# Substrate Stiffness Drives Epithelial to Mesenchymal Transition and Proliferation through the *NEAT1*-Wnt/β-Catenin Pathway in Liver Cancer

**DOI:** 10.3390/ijms222112066

**Published:** 2021-11-08

**Authors:** Xichao Xu, Yi Zhang, Xing Wang, Shun Li, Liling Tang

**Affiliations:** 1 Key Laboratory of Biorheological Science and Technology, Ministry of Education, College of Bioengineering, Chongqing University, Chongqing 400044, China; xichaoxu0@163.com (X.X.); zhangyi1@cqu.edu.cn (Y.Z.); 20116979@cqu.edu.cn (X.W.); 2 Department of Immunology, School of Basic Medical Sciences, Chengdu Medical College, Chengdu 610500, China; 3 Non-Coding RNA and Drug Discovery Key Laboratory of Sichuan Province, Chengdu Medical College, Chengdu 610500, China

**Keywords:** substrate stiffness, lncRNA *NEAT1*, WNT/β-catenin, EMT

## Abstract

Background: Extracellular matrix (ECM)-derived mechanical stimuli regulate many cellular processes and phenotypes through mechanotransduction signaling pathways. Substrate stiffness changes cell phenotypes and promotes angiogenesis, epithelial to mesenchymal transition (EMT), and metastasis in tumors. Enhanced liver tissue matrix stiffness plays a crucial role in the tumorigenesis and malignant development of liver cancer and is associated with unfavorable survival outcomes. However, how liver cancer cells sense changes in ECM stiffness and the underlying molecular mechanisms are largely unknown. Methods: Seeding HepG2 cells on the micropillar gels, HepG2 cells were assessed for responsiveness to mechanotransduction using Western blot and immunofluorescence. Conclusions: We found that higher substrate stiffness dramatically enhanced malignant cell phenotypes and promoted G1/S transition in HepG2 cells. Furthermore, nuclear paraspeckle assembly transcript 1 (*NEAT1*) was identified as a matrix stiffness-responsive long non-coding RNA (lncRNA) regulating proliferation and EMT in response to increasing matrix stiffness during the progression of HepG2 cells towards liver cancer phenotypes. Higher matrix stiffness contributed to enhancing *NEAT1* expression, which activated the WNT/β-catenin pathway. β-catenin translocates and enters the nucleus and the EMT transcription factor zinc finger E-box binding homeobox 1 (ZEB1) was upregulated to trigger EMT. Additionally, the proteins required for matrix stiffness-induced proliferation and resistance were strikingly upregulated in HepG2 cells. Therefore, our findings provide evidence that ECM-derived mechanical signals regulate cell proliferation and drive EMT through a *NEAT1*/WNT/β-catenin mechanotransduction pathway in the tumor microenvironment of liver cancer.

## 1. Introduction

Liver cancer is a main cause of cancer mortality globally. Along with chronic inflammation, fibrosis and cirrhosis can also contribute to liver cancer initiation and progression [1]. Moreover, the underlying tumorigenesis and molecular mechanisms driving liver cancer remain obscure. Thus, data on exploring the pathogenesis of liver cancer and how to improve therapeutic efficiency for liver cancer patients are urgently needed. Extracellular matrix (ECM) stiffness impacts various biological processes by activating diverse mechanotransduction pathways. In breast cancers, substrate stiffness promotes EMT and metastasis via the TWIST1-G3BP2 pathway [2]. Substrate stiffness was found to promote the expression of angiogenesis-related factors, such as VEGFA, HIF-1α, and TGF-β1, in A549 cells [3]. Clinical studies have revealed that the occurrence of liver cancer is often accompanied by cirrhosis, and the development of cirrhosis increases the risk of patients with liver cancer [4]. Increasing ECM stiffness facilitates cell proliferation and strengthens drug resistance; it also enhances integrinβ1 and phospho-FAK expression in liver cancer cells [5]. Furthermore, increasing liver matrix stiffness promotes the progression of hepatocellular carcinoma (HCC) and is correlated with unfavorable survival and prognosis [6]. Thus, liver substrate stiffness is critical for the development and progression of liver cancer. Epithelial to mesenchymal transition (EMT), which is a highly conserved and reversible cellular process, is essential for embryogenesis and cancer progression [7]. In the context of the tumor microenvironment, EMT induces cancer cell metastasis and invasion and confers malignant tumor resistance. Functionally, EMT can be activated by diverse signaling pathways, including the TGF-β, NOTCH, and WNT/β-catenin pathways [8]. Similarly, EMT can be induced in cirrhosis and liver cancer through the WNT/β-catenin pathway [9]. ECM stiffness has been found to be involved in tumor metastasis and to drive EMT by diverse mechanotransduction pathways [10,11]. Moreover, high substrate stiffness promotes the expression of N-cadherin and vimentin, as well as increases the TGF-β1-induced Smad pathway in HCC [5]. These findings suggest a role for ECM stiffness in triggering EMT during cancer metastasis. However, how ECM stiffness impacts liver cancer cellular processes and modulates liver cancer survival and metastasis and the underlying molecular mechanism are largely unknown.

Based on their length, lncRNAs are classified as ncRNAs longer than 200 bases in length [12]. Increasing evidence has demonstrated that lncRNAs can be involved in tumorigenesis, metastasis, and progression of liver cancer through modulating the liver microenvironment, and can also act as a diagnostic and therapeutic target [13,14]. Nuclear enriched abundant transcript 1 (*NEAT1*) is an important architectural component lncRNA for paraspeckle assembly and regulates the expression of several genes [15,16]. The *NEAT1* gene encodes two transcripts, *NEAT1v1* (3.7 kb) and *NEAT1v2* (23 kb) [17]. Excessive *NEAT1* expression plays a critical role in growth and resistance and is positively correlated with unfavorable survival in many cancers [18,19]. Moreover, *NEAT1* can facilitate growth, metastasis, and progression in colorectal cancer (CRC) [20] and glioma cells [21] through activating Wnt/β-catenin signaling. In addition, knockdown of *NEAT1* impedes viability and invasion in glioma cells by suppressing miRNA-132 [22]. In liver cancer cells, *NEAT1* promotes growth through modulating ATGL expression [23]. Furthermore, *NEAT1* was found to promote sorafenib-resistance via enhancing ATG3 expression and autophagy [24]. Nevertheless, the detailed molecular mechanism of *NEAT1* function in liver cancer remains elusive. *NEAT1* also regulates replication stress and drug chemosensitivity [25]. *NEAT1* was found to be significantly upregulated in response to stress in mammalian neurons [26]. In addition, *NEAT1* can be induced by HIF-2α in hypoxic stress conditions and responds to inflammasome stimuli [27]. *NEAT1* modulates the expression of IL8 through controlling paraspeckle assembly upon immune stimulation [28]. Thus, these results suggest that *NEAT1* can respond to stress, such as replication stress, hypoxic stress, and immune stimulation. These stresses can induce the expression of *NEAT1*. However, whether and how *NEAT1* senses and regulates the mechanical properties of liver cancer tissue is currently unresolved.

The ECM is composed of a complex mixture of proteins, glycosaminoglycans and proteoglycans, fibronectin, and glycoconjugate (glycoproteins) [29]. The stiffness increases with the deposition of the extracellular matrix, leading to changes in extracellular mechanical properties. Stiffness is a measure of the resistance of a material to deformation when mechanical force is on it. Material properties depend on strain, rather than on the method used to measure these properties [30]. To explore the ECM stiffness of liver cancer to impact EMT and the expression of *NEAT1*, we built 3D micropillar structure models. We demonstrated that the higher substrate stiffness could upregulate *NEAT1* expression and promote proliferation and resistance of HepG2 cells. Moreover, the higher substrate stiffness drove EMT through the WNT/β-catenin pathway to promote metastasis and proliferation. Thus, we identified the *NEAT1*-WNT/β-catenin pathway as regulating EMT and tumor metastasis in the liver cancer mechanical microenvironment.

## 2. Results

### 2.1. Higher Substrate Stiffness Enhances NEAT1 Expression in HepG2 Cells

**Figure 1:**
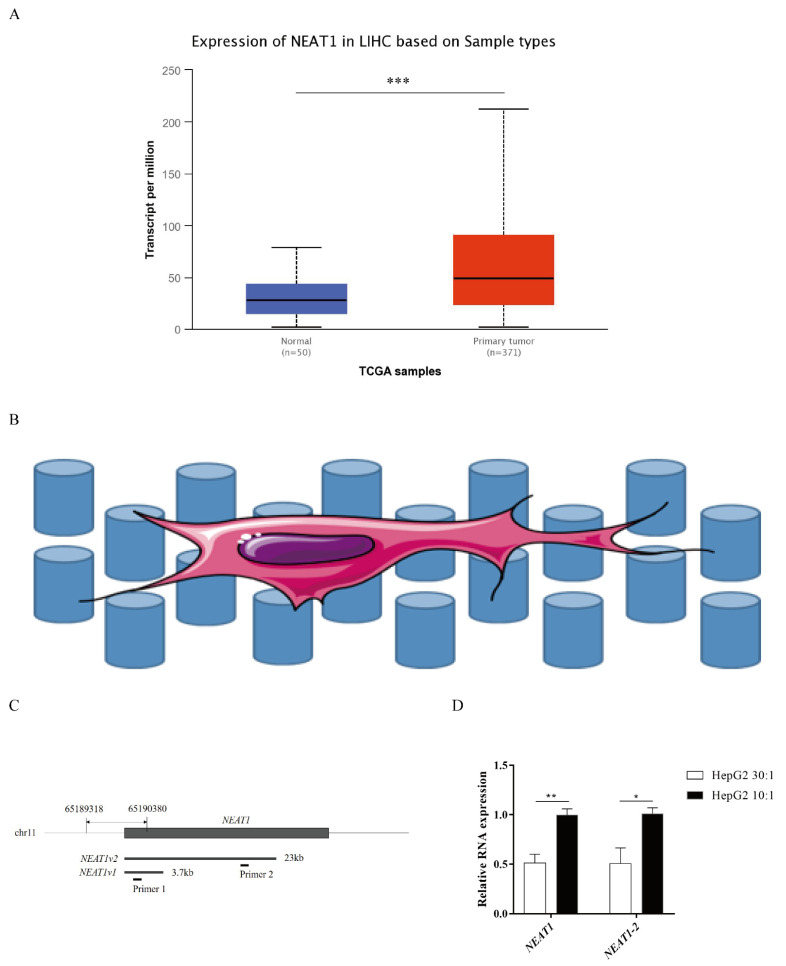
Higher substrate stiffness enhances *NEAT1* expression in HepG2 cells. (**A**) Analysis of expression of *NEAT1* in human LIHC samples compared to normal liver samples based on the TCGA using the UALCAN databases. (**B**) The model of 3D topological micropillars. The establishment of two different substrate stiffness using PDMS, and then HepG2 cells were cultured on it. (**C**) The primers of *NEAT1* and *NEAT1v2* to detect total *NEAT1* and *NEAT1v2*, respectively. (**D**) HepG2 cells are cultured on the 3D micropillars (10:1, 30:1) for 48 h. Analysis of *NEAT1* expression by qPCR. * *p* < 0.05, ** *p* < 0.01, and *** *p* <0.001.

*NEAT1* contributes to various biological processes and regulates diverse signaling pathways, especially in cancers. To determine the expression status of *NEAT1* in liver cancer, we first assessed the expression of *NEAT1* using the UALCAN database (http://ualcan.path.uab.edu/index.html, 26 September 2021). Compared with normal liver samples, the expression of *NEAT1* in LIHC primary tissue was significantly upregulated (Figure 1A). To explore how substrate stiffness impacts liver cancer cell fate and the expression of *NEAT1*, we built a model of 3D topological micropillars. We established two different stiffnesses of substrates using PDMS, including stiff substrates (10:1) and soft substrates (30:1). HepG2 cells were then cultured on these micropillars (Figure 1B). Next, we examined the expression of *NEAT1* in response to substrates by quantitative PCR (qPCR). Primer 1 identified total *NEAT1* (*NEAT1-1* and *NEAT1-2*), while primer 2 identified *NEAT1-2* only (Figure 1C). Compared with the soft substrate (30:1), both the expression levels of *NEAT1* and *NEAT1-2* were enhanced in the stiff substrate (10:1) stimulated HepG2 cells (Figure 1D), showing that substrate stiffness increased the expression levels of *NEAT1* and *NEAT1-2*. These results confirmed that *NEAT1* was upregulated in liver cancer and functioned as a substrate stiffness-associated lncRNA in HepG2 cells.

### 2.2. Higher Substrate Stiffness Regulates the Cell Cycle and Promotes Proliferation and Resistance in HepG2 Cells

**Figure 2:**
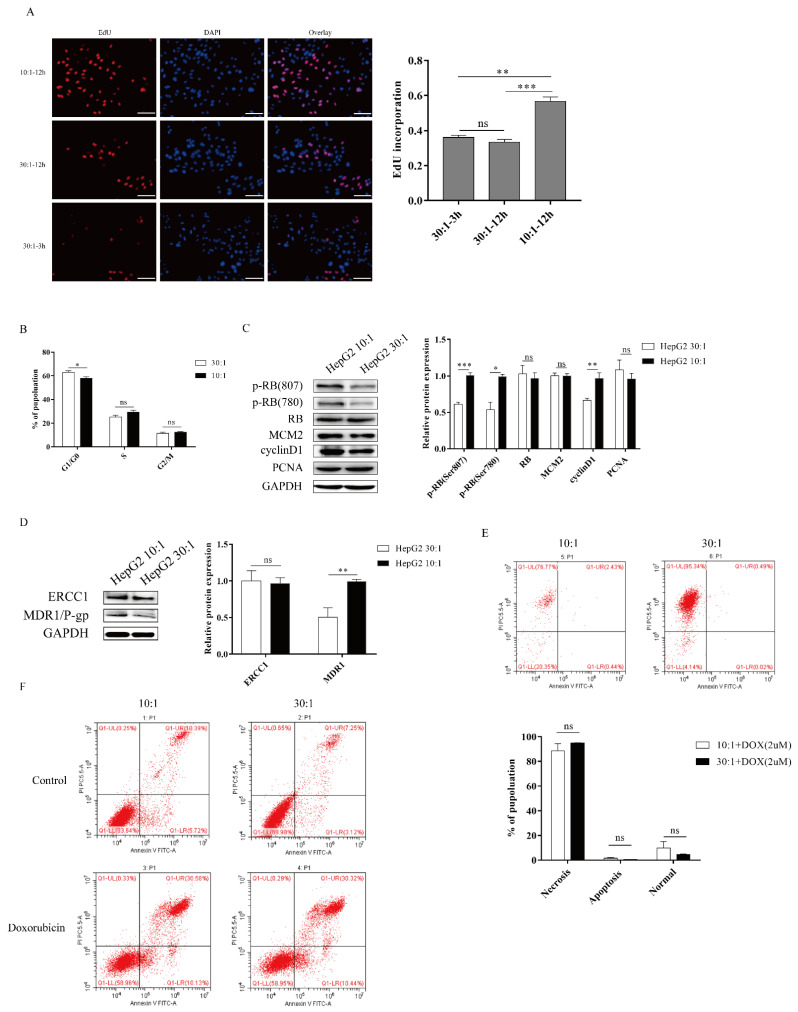
Higher substrate stiffness facilitates G1/S transition, proliferation, and resistance in HepG2 cells. (**A**) Detection of the cells proliferation after substrate stiffness treatment using EdU assay. HepG2 cells are cultured in 3D micropillars with different stiffness for 48 h before incubation with Edu. Quantification of positive cells undergoing DNA synthesis. (Scale bar: 100 μm). (**B**) Detection of the cell cycle process by flow cytometry. HepG2 cells are cultured in 3D micropillars (10:1, 30:1) for 48 h. Flow cytometry analysis shows G1, G2/M, and S populations (n = 5). (**C**) Exploration of the cell cycle-related protein expression after substrates cues stimuli in HepG2 cells. HepG2 cells were cultured on 3D micropillars (10:1, 30:1) for 48 h. Western blot analyzed the expressions of proteins. (**D**) HepG2 cells were cultured on 3D micropillars (10:1, 30:1) for 48 h. Western blot shows that resistance-related protein expression after matrix stiffness treatment. (**E**,**F**) Detection of the doxorubicin-induced cell apoptosis in HepG2 by flow cytometry. (**E**) HepG2 cells were cultured on 3D micropillars (10:1, 30:1) for 48 h. Cells were treated with 2 μM DOX. Detection of cell apoptosis was performed using flow cytometry. (**F**) HepG2 cells were cultured on 3D micropillars (10:1, 30:1) for 48 h. Cells were treated with 0.25mg/mL DOX. Detection of cell apoptosis was performed using flow cytometry. *, *p* < 0.05; **, *p* < 0.01; ***, *p* < 0.001; and ns, not significant.

To investigate the responses to ECM stiffness of HepG2 cells, we next examined the cell proliferation on different substrate stiffnesses. Using a 5-ethynyl-2′-deoxyuridine (EdU) assay, we found that DNA synthesis ability was significantly enhanced after EdU treatment on a stiff substrate compared to a soft substrate, confirming that substrate stiffness could promote the proliferation of HepG2 cells (Figure 2A). In addition, we found that the curing time of PDMS did not significantly affect the DNA synthesis ability (Figure 2A). Furthermore, the role of substrate stiffness in the cell cycle process was assessed using flow cytometry. Cell cycle analysis showed that substrate stiffness cues caused a G1/S transition, and a stiff substrate (10:1) markedly decreased the G1 population compared to a soft substrate (30:1) in HepG2 cells (Figure 2B). Thus, these results indicated that substrate stiffness promotes cell proliferation and G1/S transition in HepG2 cells. Next, we sought to explore the underlying molecular mechanism by which substrate stiffness cues regulated HepG2 cells proliferation and G1/S transition. The RB signaling pathway is a major regulator of G1/S transition. We therefore assessed whether substrate stiffness regulated the cell cycle and promoted proliferation through the RB signaling pathway. As expected, the expression of p-RB (Ser780) and p-RB (Ser807) was found to be prominently increased in response to a stiff substrate (10:1) (Figure 2C), implying that substrate stiffness induced G1/S transition in HepG2 cells through the RB signaling pathway. Moreover, we investigated the expression of other cell cycle-related proteins after substrate cue stimuli, including mini-chromosome maintenance proteins 2 (MCM2), PCNA, and cyclin D1. MCM2 and PCNA protein levels did not significantly change in response to substrate stiffness (Figure 2C). The protein level of cyclin D1 was increased in response to a stiff substrate (10:1) (Figure 2C). To study cell apoptosis in response to substrate stiffness, we assessed the level of doxorubicin-induced cell apoptosis in HepG2 cells by flow cytometry. Flow cytometry analysis revealed that substrate stiffness cues did not affect doxorubicin-induced cell apoptosis in HepG2 cells under different concentrations of doxorubicin (Figure 2E,F). We also found that higher substrate stiffness could accelerate the expression of MDR1/P-gp. However, substrate stiffness did not affect the expression of ERCC1, suggesting that stiff substrate contributes to drug resistance through boosting drug efflux (Figure 2D). Therefore, these results indicated that higher matrix stiffness could facilitate cell proliferation, G1/S transition, and resistance in HepG2 cells.

### 2.3. Matrix Stiffness Signals Promote EMT through Activating the WNT/β-Catenin Pathway

**Figure 3:**
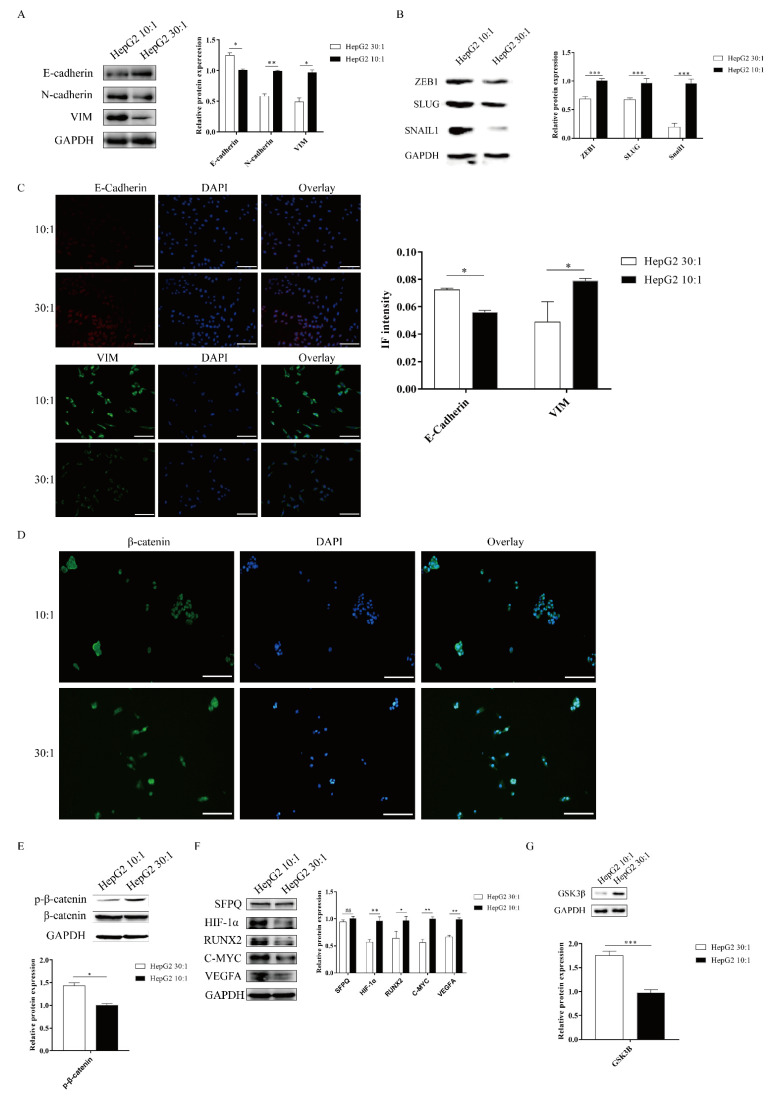
Matrix stiffness signals promote EMT through activating the WNT/β-Catenin pathway. (**A**–**C**) Effect of substrate stiffness on the EMT phenotype. HepG2 cells are cultured on 3D micropillars (10:1, 30:1) for 48 h. (**A**,**B**) Western blot and (**C**) IF analysis of phenotypic markers and TF. (Scale bar: 100 μm). (**D**–**G**) Effect of substrate stiffness on the WNT/β-Catenin pathway. (**D**) Investigation of the localization of β-Catenin by IF in HepG2 cells. HepG2 cells were cultured in 3D micropillars (10:1, 30:1) for 72 h. IF analysis of β-Catenin localization. (Scale bar: 100 μm). (**E**) HepG2 cells were cultured in 3D micropillars (10:1, 30:1) for 48 h. Western blot analyzed the expression of p-β-Catenin and β-Catenin. (**F**) HepG2 cells were cultured in 3D micropillars (10:1, 30:1) for 48 h. Western blot analyzed the expressions of the downstream of WNT/β-Catenin protein. (**G**) HepG2 cells were cultured in 3D micropillars (10:1, 30:1) for 48 h. Western blot analysis of the expression of GSK3β. *, *p* < 0.05; **, *p* < 0.01; ***, *p* < 0.001; and ns, not significant.

ECM signals from the tumor microenvironment can regulate the EMT process. Matrix stiffness has been shown to drive EMT and promote breast cancer metastasis via diverse mechanotransduction signaling pathways [2,10]. However, whether matrix stiffness triggers EMT and promotes tumor metastasis in liver cancer remains unclear. To examine the mechanical responsiveness of the EMT process in HepG2 cells, we measured the expression of EMT makers via Western blot and cell immunofluorescence. Western blotting analysis revealed that the stiff substrate (10:1) significantly enhanced the expression of the mesenchymal markers N-cadherin and vimentin and remarkably decreased the expression of the epithelial marker E-cadherin (Figure 3A). We also found that SNAIL1, SLUG, and ZEB1, transcription factors associated with EMT, were significantly upregulated on a stiff substrate compared to a soft substrate (Figure 3B). Meanwhile, IF staining analysis also demonstrated that higher matrix stiffness promoted mesenchymal phenotypes, including upregulated the expression of vimentin and reduced E-cadherin expression (Figure 3C). Therefore, these results indicated that higher matrix stiffness could drive EMT in HepG2 cells.

The WNT/β-catenin pathway plays an important role in the EMT process [29]. We next explored how the WNT/β-catenin pathway responds to substrate stiffness in HepG2 cells. The activation of the WNT/β-catenin pathway depends on the localization of β-catenin. To study the activation of the WNT/β-catenin pathway in HepG2 cells, we performed β-catenin IF staining to assess the localization in HepG2 cells. We found that more β-catenin staining signal colocalized with DAPI, which marks the nucleus, on a stiff substrate stiffness compared to a soft substrate (Figure 3D), suggesting that high matrix stiffness facilitates β-catenin translocation into the nucleus. To further confirm that high substrate stiffness could activate the WNT/β-catenin pathway in HepG2 cells, we determined the phosphorylation levels of β-catenin by immunoblotting. Compared with a soft substrate, a high matrix stiffness caused a marked reduction in the expression of phosphorylated β-catenin (Figure 3E). We also detected the downstream targets of the WNT/β-catenin pathway in HepG2 cells after the stiff substrate treatment, including HIF-1α, RUNX2, C-MYC, and VEGFA. Results demonstrated that a stiff substrate could significantly elevate the expressions of the downstream targets of the WNT/β-catenin pathway in HepG2 cells compared to a soft substrate (Figure 3F). GSK3β is upstream of β-catenin and can combine with p-β-catenin to promote β-catenin degradation. Compared to a soft substrate, high substrate stiffness could significantly repress GSK3β expression (Figure 3G). Taken together, these results indicated that high substrate stiffness promoted EMT through activating the WNT/β-catenin pathway in HepG2 cells.

### 2.4. The lncRNA NEAT1 Activates the WNT/β-Catenin Pathway in Response to Matrix Stiffness Stimuli

**Figure 4:**
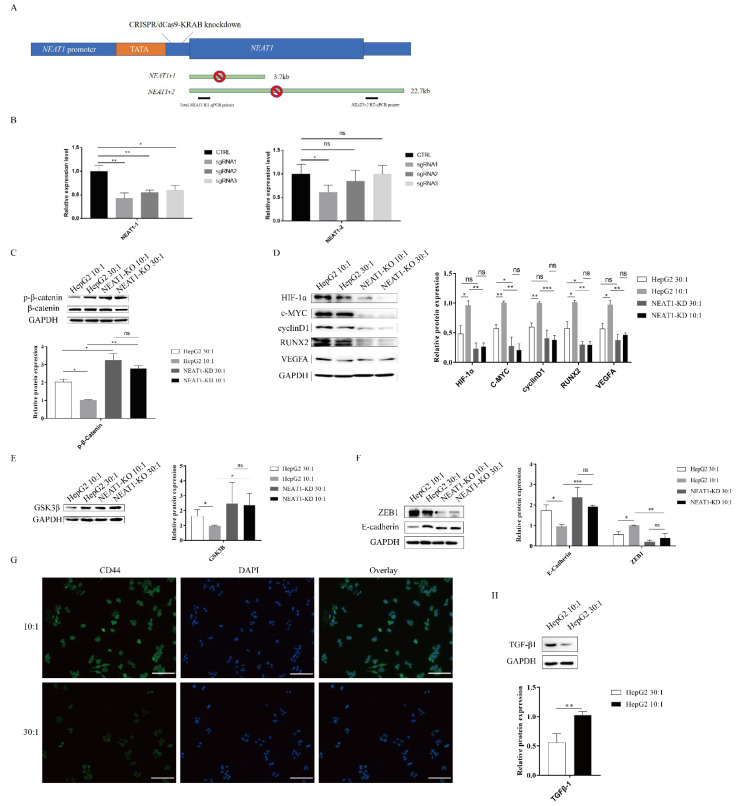
The lncRNA *NEAT1* activates the WNT/β-catenin pathway in response to matrix stiffness stimuli. (**A**,**B**) Generation of HepG2 cell lines lacking expression of *NEAT1* by CRISPR/dCas9-KRAB. (**A**) A schematic representation of the total *NEAT1* knockdown strategy through CRISPR/dCas9-KRAB-mediated knockdown. A dCas9-KRAB guided by either *NEAT1* promoter-targeting sgRNA was introduced to the region between the TATA box and the transcription start site (TSS) of *NEAT1*. (**B**) *NEAT1* knockdown efficiency was detected by the two RT-qPCR primers (black bars). (**C**–**E**) Effect of substrate stiffness on the expression of β-catenin in HepG2-KD and HepG2 cells. (**C**) HepG2 cells were cultured in 3D micropillars (10:1, 30:1) for 24 h. Western blot analyzed the protein expressions of β-catenin and p- β-catenin. (**D**) Effects of substrate stiffness on the expression of the downstream protein of Wnt/β-Catenin in HepG2-KD and HepG2 cells. HepG2 cells were cultured in 3D micropillars (10:1, 30:1) for 24 h. Western blot analysis of protein expressions. (**E**) Effects of substrate stiffness on the expression of GSK3β in HepG2-KD and HepG2 cells. HepG2 cells were cultured in 3D micropillars (10:1, 30:1) for 24 h. Western blot analysis of protein expressions. (**F**) Effects of substrate stiffness on the protein expressions of EMT in HepG2-KD and HepG2 cells. HepG2 cells were cultured in 3D micropillars (10:1, 30:1) for 24 h. Western blot analyzed the expression of E-cadherin and ZEB1. (**G**) Effects of substrate stiffness on the expression of CD44. HepG2 cells were cultured in 3D micropillars (10:1, 30:1) for 72 h. IF analysis of CD44 expression. (Scale bar: 100 μm). (**H**) HepG2 cells were cultured on 3D micropillars (10:1, 30:1) for 24 h. Western blot analyzed the expression of TGF-β1. *, *p* < 0.05; **, *p* < 0.01; ***, *p* < 0.001; and ns, not significant.

As mentioned above, high substrate stiffness could upregulate *NEAT1* expression (Figure 1C) and activate the WNT/β-catenin pathway (Figure 3). We therefore speculated that matrix stiffness activates the WNT/β-catenin pathway through upregulating *NEAT1* expression in HepG2 cells. To test this hypothesis, we first knocked down *NEAT1* expression using CRISPR/dCas9-KRAB (Figure 4A). The efficiency of *NEAT1* knockdown was verified by qPCR using two different PCR primers. CRISPR/dCas9-KRAB-sgRNA1 significantly decreased the expressions of total *NEAT1* and *NEAT1-2* (Figure 4B). Thus, we chose HepG2-sgRNA1 for the following studies. In support of our hypothesis, we found that inhibition of *NEAT1* expression remarkably strengthened the expression of phosphorylated β-catenin in *NEAT1*-KD cell lines compared with controls, while matrix stiffness cues had no impact on the phosphorylation levels of β-catenin in *NEAT1*-KD cell lines (Figure 4C). To investigate the effects of *NEAT1*-KD on the activation of the WNT/β-catenin pathway upon stiffness stimuli, we next monitored the downstream targets of the WNT/β-catenin pathway in *NEAT1*-KD HepG2 and *NEAT1*-WT HepG2 cells. Impeding the expression of *NEAT1* could restrain the downstream targets of the WNT/β-catenin pathway, including HIF-1α, c-MYC, RUNX2, cyclin D1, and VEGFA (Figure 4D). However, there were no significant differences in the downstream targets of the WNT/β-catenin pathway between a stiff substrate and a soft substrate after *NEAT1* inhibition (Figure 4D). We also determined the expression of GSK3β after matrix stiffness treatment in *NEAT1*-KD HepG2 and *NEAT1*-WT HepG2 cells. Results showed that the inhibition of *NEAT1* could enhance GSK3β expression compared with controls (Figure 4E). To further explore the role of *NEAT1* in EMT, we examined E-cadherin and ZEB1 expression after matrix stiffness treatment in *NEAT1*-KD HepG2 cells. We found that the expression of the epithelial marker E-cadherin was upregulated in *NEAT1*-KD HepG2 cells compared with controls. Contrary to this, inhibition of *NEAT1* could attenuate the expression of ZEB1 (Figure 4F), resulting in a disruption of the EMT process. Therefore, these results demonstrated that matrix stiffness modulates the WNT/β-catenin pathway through upregulating *NEAT1* expression and then triggers EMT.

HIF-1α, VEGFA, and TGF-β1 are important factors for tumor angiogenesis. As shown in Figure 3 and Figure 4, high matrix stiffness can contribute to the expression of HIF-1α and VEGFA through the expression of *NEAT1*. We therefore explored whether high matrix stiffness enhanced TGF-β1 expression. As expected, high matrix stiffness could induce the expression of TGF-β1 (Figure 4G). CD44 and RUNX2, as well as the EMT process, play a critical role in tumor metastasis. We also assessed the expression of CD44, which may serve as a metastasis and angiogenesis factor in tumors. The expression of CD44 was found to be upregulated after high matrix stiffness treatment (Figure 4H).

### 2.5. The lncRNA NEAT1 Regulates the EMT Process through YB1

**Figure 5:**
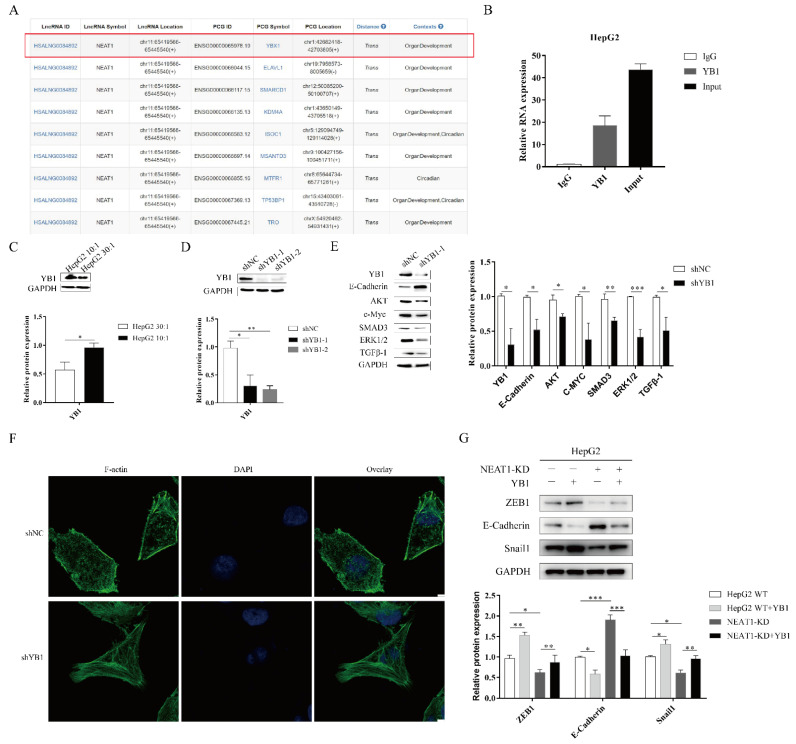
The lncRNA *NEAT1* regulates the EMT process through YB1. (**A**) Analysis of the RNA binding proteins (RBPs) using an online database. We list relative score > 80% of RBP (YBX1). (**B**) The RNA immunoprecipitation experiment was performed using YB1 and IgG antibodies to probe HepG2 cell extracts, and the level of *NEAT1* expression was determined using qPCR. (**C**) Effects of substrate stiffness on the expression of YB1 in HepG2 cells. HepG2 cells were cultured in 3D micropillars (10:1, 30:1) for 48 h. (**D**,**E**) The silencing efficiency of YB1 and downstream target in HepG2 cells were detected using WB. (**F**) HepG2 cells were treated after lentivirus transduction for 72 h. Phalloidin analysis of F-actin expression and localization by confocal microscope. (Scale bar: 5 μm). (**G**) Effects of YB1 on the expression of EMT-associated protein in HepG2-KD and HepG2 cells. Transfection of YB1 plasmid in HepG2-KD and HepG2 cells for 72 h. Western blot analyzed the protein expressions of E-Cadherin, ZEB1, and Snail1. *, *p* < 0.05; **, *p* < 0.01; and ***, *p* < 0.001.

To further investigate the function of *NEAT1* in the progression of liver cancer, we analyzed RNA binding proteins (RBPs) using an online database. Fortuitously, based on the RBPDP database [30], we found that *NEAT1* could bind to the RNA binding protein YB1 (Figure 5A). The website RNA-Protein Interaction Prediction (RPISeq) also showed an interaction between YB1 and *NEAT1*. The score of the interaction probabilities was 0.85. To validate the interaction between *NEAT1* and YB1, we next performed an RNA-IP assay. As shown in Figure 5B, *NEAT1* was markedly enriched in HepG2 cells using a YB1-specific antibody compared to an IgG group in HepG2 cells. These results demonstrated that *NEAT1* bound the YB1 protein.

To assess whether YB1 was involved in mechanotransduction in liver cancer, we detected the expression of YB1 upon mechanical stimulation. We found that higher matrix stiffness augmented the expression of YB1 compared with a soft substrate (Figure 5C). To study the function of YB1, we generated an shRNA-target YB1 vector and overexpression YB1 vector. The silencing of YB1 significantly diminished the protein expression of YBX1 (Figure 5D). Subsequently, YB1 knockdown enhanced E-cadherin expression and attenuated the expression of downstream targets, including AKT, c-MYC, ERK1/2, and TGF-β1 (Figure 5E). We next sought to investigate whether YB1 modulated the mechanical properties of HepG2 cell lines. We also performed phalloidin analysis of F-actin in HepG2-YB1 knockdown cells. In the vector control group, F-actin was sparse and disorderly arranged. However, F-actin in the YB1 knockdown group was more orderly and dense (Figure 5F). To identify how and whether the interaction between *NEAT1* and YB1 contributed to liver cancer, we overexpressed YBX1 in *NEAT1*-KD HepG2 cell lines. Compared to the control group, the overexpression of YB1 partially restored the expression of Snail1 and ZEB1 while decreasing the expression of E-Cadherin in *NEAT1*-silenced HepG2 cells (Figure 5G). These results indicated that YB1 participated in the mechanical properties of HepG2 cells. In conclusion, *NEAT1* regulated the EMT process by binding to YB1 in liver cancer.

### 2.6. The lncRNA NEAT1 Promotes the Growth of Liver Cancer In Vivo

**Figure 6:**
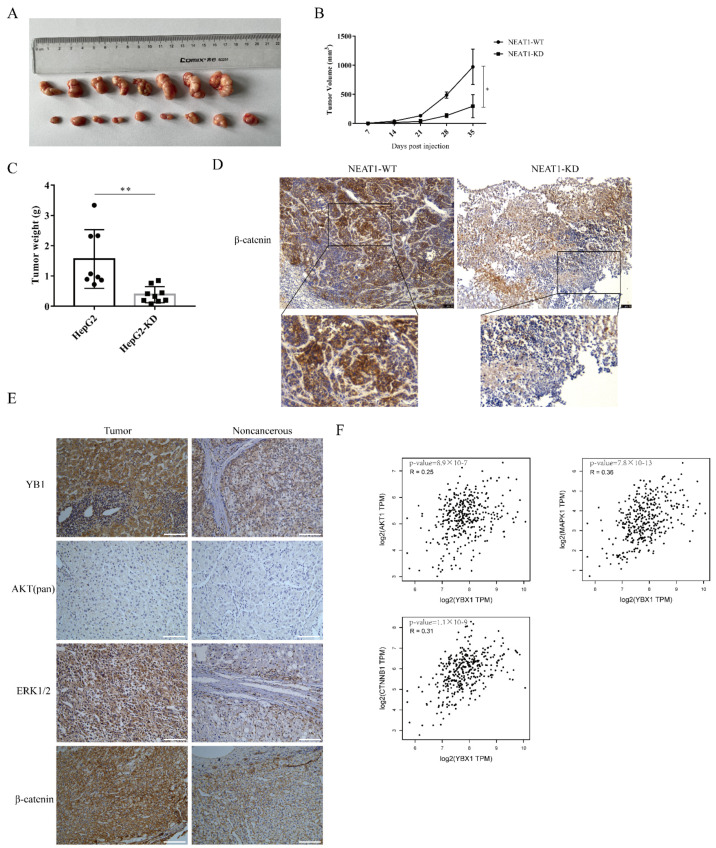
The lncRNA *NEAT1* promotes the growth of liver cancer in vivo. *NEAT1*-KD HepG2 cells were inoculated into the subcutaneous tissue of nude mice. The tumor picture (**A**), the tumor volume (**B**), and tumor weight (**C**) are shown and compared among the groups; * is *p* < 0.05 and ** is *p* < 0.01. (**D**) Immunohistochemistry (IHC) staining of β-catenin in the tumors. The activation of the WNT/β-catenin pathway depends on the localization of β-catenin. The IHC staining of β-catenin overlay with the nuclear was displayed below. Scale bar: 75 μm. (**E**) IHC analysis to detect the expression of YB1, AKT, ERK, and β-catenin in liver cancer samples from patients. Scale bar: 100 μm. (**F**) The correlation between YB1 and AKT, ERK, and β-catenin in LIHC from the GEPIA database. The R values and *p* values are from Pearson’s correlation analysis.

To further examine the role of *NEAT1* in regulating the growth of liver cancer in vivo, *NEAT1*-silenced HepG2 cells were inoculated into the subcutaneous tissue of nude mice. Consistent with our observations in vitro, silencing of *NEAT1* significantly attenuated tumor volumes (Figure 6A,B) and mitigated tumor weight relative to the control group (Figure 6C), suggesting that silencing of *NEAT1* inhibits liver cancer growth. Immunohistochemistry (IHC) staining revealed that the activation of the β-catenin pathway was markedly decreased in tumors with *NEAT1* knockdown (Figure 6D). These results confirmed that *NEAT1* knockdown significantly diminished liver cancer cell growth and proliferation and inhibited the activation of the β-catenin pathway in vivo. To investigate whether the results above were related to clinical patients, we examined the expression of β-catenin, YB1, AKT, and ERK protein in liver cancer samples. IHC staining showed that the protein expression of β-catenin, YB1, AKT, and ERK was remarkably higher in tumor samples than in tumor-adjacent tissue from patients (Figure 6E). Importantly, an analysis of the Pearson correlation coefficient indicated a positive correlation between YB1 and AKT, ERK, and β-catenin in liver cancer (Figure 6F), implying the important role of YB1 in regulating LIHC tumorigenesis in vivo.

## 3. Discussion

Liver cancer is the third cause of cancer mortality worldwide and has been linked with increased cancer death rates in recent years [31]. The development of most liver cancers is based on liver cirrhosis, and patients with cirrhosis have poor outcomes [4,32]. Previously, a substantial number of studies have revealed the vital roles of ECM stiffness on metastasis in tumors [2,10,33]. Moreover, ECM is involved in tumor progression and impacts the EMT process [34]. However, how ECM stiffness impacts liver cancer metastasis and the underlying molecular mechanisms remain unresolved. Thus, this study aimed to explore how *NEAT1* senses tissue stiffness and regulates liver cancer metastasis. In this study, we found that higher substrate stiffness upregulated *NEAT1* expression. The stiff substrate promoted proliferation and EMT through activating WNT/β-catenin in HepG2 cells. Our findings also suggested that *NEAT1* was associated with mechanotransduction in liver cancer.

A previous study showed that *NEAT1* could regulate the stability of DDX5 and then activate the WNT/β-catenin pathway [20]. Recent studies have also indicated that *NEAT1* activates the WNT/β-catenin pathway through binding to EZH2 and then facilitates cancer progression [21]. However, whether *NEAT1* regulates the WNT/β-catenin pathway upon mechanical stimuli remains unknown. Our results have shown that *NEAT1* knockdown promotes β-catenin phosphorylation and attenuates WNT/β-catenin pathway activation, resulting in disrupting the downstream target expressions of the WNT/β-catenin pathway. *NEAT1* knockdown also reduces ZEB1 expression and increases the expression of E-cadherin, thereby disrupting the EMT process. A higher matrix stiffness also decreased the expression of GSK3β. *NEAT1* knockdown enhanced the expression of GSK3β in HepG2 cells. GSK3β regulates the WNT/β-catenin pathway through phosphorylation of beta-catenin (Ser33/37/Thr41) [35]. Additionally, the phosphorylation of GSK3β correlates with its activity. Thus, whether *NEAT1* activates the WNT/β-catenin pathway through GSK3β still needs to be resolved. We also found that high substrate stiffness upregulated the expression of MDR1/p-gp. However, high substrate stiffness did not alter the sensitivity of HepG2 cells to doxorubicin. Thus, the underlying molecular mechanisms need further study.

There have been several reports about lncRNAs being involved in the mechanical properties of cells. Soft substrate upregulates lncRNA *LINC00458* expression and impacts the differentiation of hPSC [36]. *LINC00472* can regulate the EMT process and affect the mechanical properties of lung adenocarcinoma cells via binding to YB1 [37]. *LINC01569* directly binds to YB1 and regulates mechanosensors [38]. Nonetheless, this is the first report showing that *NEAT1* participates in mechanotransduction in liver cancer. In this study, we also found that the RNA binding protein YB1 could interact with *NEAT1*. High matrix stiffness enhanced the expression of YB1 in HepG2 cells. However, how YB1 regulates the progression of liver cancer upon mechanical stimuli needs further study.

**Figure 7:**
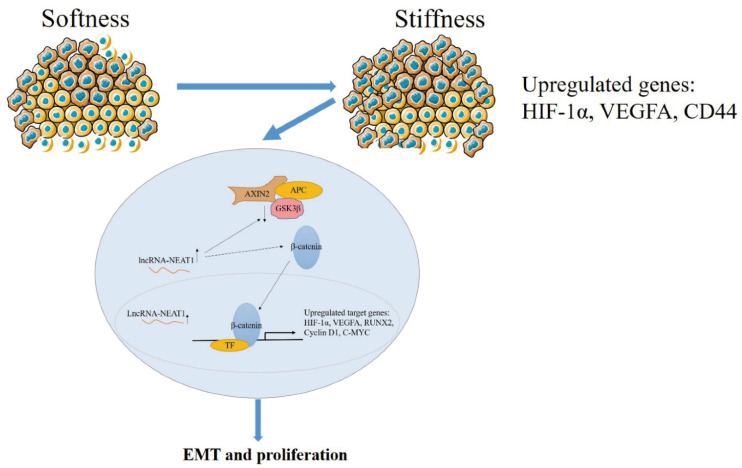
The schematic diagram of the mechanism for substrate stiffness regulated *NEAT1* function in HepG2 cells.

In general, higher substrate stiffness modulates the cell cycle and strengthens *NEAT1* expression in HepG2 cells. Enhanced *NEAT1* expression can activate the WNT/β-catenin signaling pathway, and then the WNT/β-catenin signaling pathway contributes to the EMT process and promotes tumor proliferation in liver cancer (Figure 7). This study elucidated the mechanisms of substrate stiffness-induced *NEAT1* regulation of the EMT process through activating the WNT/β-catenin pathway in HepG2 cells, suggesting that the *NEAT1*-WNT/β-catenin pathway may potentially serve as a target for the clinical therapy of liver cancer.

## 4. Materials and Methods

### 4.1. Cell Culture

HepG2 cells, obtained from Professor Jun Zhang from Chongqing Medical University, were grown in minimum essential medium (BasalMedia, Shanghai, China) supplemented with 10% FBS (LONSERA, Canelones, Uruguay) and 1% penicillin and streptomycin. The Hep G2 cells were a hepatoblastoma-derived cell line. They were not derived from a classical hepatocellular carcinoma (HCC). Additionally, 293FT cells were cultured in Dulbecco’s Modified Eagle’s Medium (BasalMedia, Shanghai, China) and supplemented with 10% FBS and 1% penicillin and streptomycin.

### 4.2. Preparation of 3D Topological Micropillar Gels of Different Stiffnesses In Vitro

In brief, a polydimethylsiloxane substrate SYLGARDTM 184 Silicone Elastomer Kit (PDMS, Midland, MI, USA), comprising two components, a silicone elastomer and a silicone elastomer curing agent, were used. For the preparation of 10:1 (higher stiff substrate) and 30:1 (soft substrate) PDMS gels, silicone elastomer, and the curing agent were thoroughly mixed together. The mixture was placed into a mold for a 3D topological micropillar, and then cross-linked on a heater at 80 °C for 12 h. PDMS substrate gels were next sterilized using UV for 2 h to overnight, and then incubated of fibronectin (Fn, Millipore, Billerica, USA) at 37 °C for 2 h or 4 °C overnight to facilitate cell adhesion. HepG2 cells were then seeded on the micropillar gels and cultured at 37°C in 5% CO_2_ for 48 h. Next, the HepG2 cells were harvested when the cell density reached approximately 90%, and then the cells were lysed using lysis buffer (Solarbio, Beijing, China).

### 4.3. Plasmid Construction, Lentivirus, sgRNAs, and Transfection

For *NEAT1* knockdown using CRISPR/dCas9-KRAB, guide RNAs were cloned into lentiGuide-Puro (Addgene #52963). For YBX1 knockdown using shRNA, shRNAs were cloned into the pLKO.1-TRC cloning vector (Addgene #10878). For overexpression of YBX1, the coding sequence of YBX1 was cloned into pLVX-DsRed-Monomer-N1 (Takara, Kyoto, Japan). The sequences of sgRNAs and primers are listed in Table S1. Stable *NEAT1* and YBX1 knockdown and overexpression of YBX1 HepG2 cells were generated using lentiviral plasmids. In brief, to knock down *NEAT1*, 293FT cells were transfected with pCMV-dR8.2 dvpr, pCMV-VSV-G, lentiGuide-Puro, and dCas9-KRAB (Addgene #110820). To knock down YBX1, 293FT cells were transfected with pCMV-dR8.2 dvpr, pCMV-VSV-G, and pLKO.1-shRNAs. Viral supernatants were harvested, and then HepG2 cells were infected with viral particles. These HepG2 cells were then selected to generate stable clones with puromycin (2 μg/mL).

### 4.4. RNA Extraction, Reverse Transcription, and Quantitative Real-Time Polymerase Chain Reaction (qRT-PCR) Analysis

Total RNA from HepG2 cells was isolated using RNAiso Plus (Takara). cDNA was synthesized using the PrimeScript RT Mix (Takara) kit according to the manufacturer’s instructions. Quantitative real-time PCR was performed using the NovoStart SYBR qPCR SuperMix Plus kit (novoprotein, Suzhou, China). *NEAT1* gene expression was normalized to GAPDH and relative quantification was determined using the 2^−∆∆Ct^ method. The sequences of primers are listed in Table S1.

### 4.5. Western Blotting Analysis

HepG2 cells were lysed using lysis buffer (Solarbio, China), proteins (40 μg) were loaded into 10% or 12% gels (Solarbio, China) and then transferred onto nitrocellulose membranes. After blocking, the membranes were cropped according to the molecular weight marker and the molecular weight of the target protein on the primary antibody instruction. Then, the membranes were incubated with primary antibodies overnight at 4 °C. The next day, the membranes were washed three times with TBST buffer for 15 min and incubated with secondary antibodies for 1–2 h at room temperature. Then, the membranes were washed three times with TBST buffer for 30 min. Finally, we used Azure Biosystems to acquire blot images. If the molecular weight of the different proteins were similar, the membranes were washed three times with TBST for 30 min and incubated with 5 mL stripping buffers (ThermoFisher, Waltham, MA, USA) for 10–15 min after ECL development. Then, the membranes were washed three times with TBST for 15 min. After reblocking, membranes were incubated with a different primary antibody overnight at 4 °C according to the manufacturer’s recommendations and the molecular weight of the target protein. The primary antibodies used are listed in Table S2.

### 4.6. Flow Cytometry Analysis

Flow cytometry was performed using a kit (Beyotime, Shanghai, China) to detect the cell cycle and cell apoptosis according to the instructions.

### 4.7. Immunofluorescence and EdU Assay

HepG2 cells were fixed in 4% paraformaldehyde at room temperature for 15 min, and these HepG2 cells were blocked with 5% BSA containing 0.3% TritonX-100 buffer at room temperature for 60 min. The primary antibodies used are listed in Table S2. Secondary antibodies containing either FITC or Cy3 (Beyotime, Shanghai, China) were used using immunofluorescence staining. An inverted microscope was used to analyze cell staining. For cell proliferation analysis, HepG2 cells were processed using the BeyoClickTM EdU Cell Proliferation Kit with Alexa Fluor555 (Beyotime, Shanghai, China) kit according to the instructions.

### 4.8. Immunohistochemistry

Paraffin-embedded liver cancer samples from patients and cancer tissue from nude mice were immunostained with antibodies after deparaffinization and hydration. The signal of IHC was amplified via a kit (#PV-9001, Zhongshan Golden Bridge Biotechnology, Beijing, China). Endogenous peroxidase was blocked with 3% hydrogen peroxide. Tissue sections were then incubated with β-catenin, YB1, AKT (pan), and ERK1/2 antibodies overnight at 4 °C. The next day, tissue sections were incubated with 100 μL or an appropriate amount of reaction enhancement solution at room temperature for 20 min. Tissue sections then received 100 μL or an appropriate amount of enhanced enzyme-labeled goat anti-rabbit IgG polymer and incubated at room temperature for 20 min. Antibody binding was assessed with a DAB kit (#ZLI-9019, Zhongshan Golden Bridge Biotechnology, Beijing, China). Then, tissue sections were counter-stained with hematoxylin. The pictures of immunostaining were captured by a microscope.

### 4.9. Animal Experiments

Nude mice were purchased from GemPharmatech (Chengdu, China). All animal experiments were performed according to Chongqing University Animal Care Guidelines. For subcutaneous xenografts, HepG2 cells stably silencing *NEAT1* (5 × 10^6^) were injected into 6-week-old female nude mice. Tumor length (L) and width (W) were monitored weekly. Tumor volume was calculated through (L × W2)/2 [39]. Tumors were then harvested and fixed in 4% PFA.

## Data Availability

All data generated or analyzed during this study are included in this published article [and its Supplementary Information Files].

## Supplementary Materials

The following are available online at https://www.mdpi.com/article/10.3390/ijms222112066/s1.
