# Supplementary material for: Substrate Stiffness Drives Epithelial to Mesenchymal Transition and Proliferation through the NEAT1-Wnt/β-Catenin Pathway in Liver Cancer
Source: Int J Mol Sci. 2021 Nov 8;22(21):12066. doi: 10.3390/ijms222112066 (PMC8584463; doi:10.3390/ijms222112066)
Supplement: Supplementary file 1 [file ijms-22-12066-s001.zip › ijms-1383051-supplementary.pdf]

Supplementary information

Table S1. The primers in this paper

REAGENT or RESOURCE	SOURCE	IDENTIFIER
Primers		
**NEAT1-F:**<br>5'GTGGCTGTTGGAGTCGGTAT3'	This paper	N/A
**NEAT1-R:**<br>5'TAACAAACCACGGTCCATGA3'	This paper	N/A
**NEAT1_2F:**<br>5'GTACTCTGTGATGGGGTAGTCA GTCAG3'	This paper	N/A
**NEAT1_2R:**<br>5'GTACTCTGTGATGGGGTAGTCA GTCAG3'	This paper	N/A
**GAPDH-F:**<br>5'GGAGCGAGATCCCTCCAAAAT3'	This paper	N/A
**GAPDH-R:**<br>5' GGCTGTTGTCATACTTCTCATGG 3'	This paper	N/A
**NEAT1 sgRNA1 targeting sequence:**<br>sgRNA1-F: 5'GGCTATAAAAGCAAAAGTTG3'<br>sgRNA1-R: 5'CAACTTTTGCTTTTATAGCC3'	This paper	N/A
**NEAT1 sgRNA2 targeting sequence:**<br>sgRNA2-F: 5'GGTCCAGCCGGAGTTAGCGA3'<br>sgRNA2-R: 5'TCGCTAACTCCGGCTGGACC3'	This paper	N/A
**NEAT1 sgRNA3 targeting sequence:**<br>sgRNA3-F: 5'GATTGCCTTCATAACGACTT3'<br>sgRNA3-R: 5'AAGTCGTTATGAAGGCAATC3'	This paper	N/A
**YB1 shRNA sequence:**<br>**shRNA #1**<br>5'-	This paper	N/A

5’-CCGGAGCAGACCGTAACCATTATAGCTCGAGCTATAATGGTTACGGTCTGCTTTTTTG-3’

5’-AATTCAAAAAAGCAGACCGTAACCATTATAGCTCGAGCTATAATGGTTACGGTCTGCT-3’

shRNA #2

5’-CCGGCCAGTTCAAGGCAGTAAATATCTCGAGATATTTACTGCCTTGAACTGGTTTTTG-3’

5’-AATTCAAAAACCAGTTCAAGGCAGTAAATATCTCGAGATATTTACTGCCTTGAACTGG-3’

shRNA #3

5’-CCGGGACGGCAATGAAGAAGATAAACTCGAGTTTATCTTCTTCATTGCCGTCTTTTTG-3’

5’-AATTCAAAAAGACGGCAATGAAGAAGATAAACTCGAGTTTATCTTCTTCATTGCCGTC-3’

Table S2. The antibodies in this paper

REAGENT or RESOURCE	SOURCE	IDENTIFIER
Antibodies		
GAPDH (human)	Proteintech	Cat#10494-1-AP
N-Cadherin (human)	Cell Signaling Technology	Cat#13116
E-Cadherin (human)	Cell Signaling Technology	Cat#3195
Vimentin	Santa Cruz Biotechnology	Cat#sc-373717
Slug (human)	Cell Signaling Technology	Cat#9585
CD44 (human)	Cell Signaling Technology	Cat#3570
VEGFA (human)	Abcam	Cat#ab1316
c-Myc (human)	Cell Signaling Technology	Cat#18583
Cyclin D1 (human)	Cell Signaling Technology	Cat#2978
PCNA (human)	Cell Signaling Technology	Cat#2586
β-Catenin (human)	Cell Signaling Technology	Cat#8480
p-β-Catenin (Ser675) (human)	Cell Signaling Technology	Cat#4176
RUNX2 (human)	Cell Signaling Technology	Cat#12556
HIF-1α (human)	Cell Signaling Technology	Cat#36169
p-Rb (Ser780) (human)	ZENBIO	Cat#380879
p-Rb (Ser807) (human)	ZENBIO	Cat#382889
RB1 (human)	Proteintech	Cat#25628-1-AP

MCM2 (human)	ZENBIO	Cat#220635
GSK3β (human)	ZENBIO	Cat#200494-2E6
ZEB1 (human)	ZENBIO	Cat#220860
MDR1/P-gp (human)	Proteintech	Cat#22336-1-AP
ERCC1 (human)	Proteintech	Cat#14586-1-AP
YB1	Cell Signaling Technology	Cat#4202
AKT	Cell Signaling Technology	Cat#4691
ERK1/2	Cell Signaling Technology	Cat#4695
β-Catenin	Cell Signaling Technology	Cat#8480
SNAIL1	Cell Signaling Technology	Cat#3879
SFPQ	Proteintech	Cat#15585-1-AP
TGF-β1	Proteintech	Cat#21898-1-AP
